# A Culturally Relevant Smartphone-Delivered Physical Activity Intervention for African American Women: Development and Initial Usability Tests of Smart Walk

**DOI:** 10.2196/15346

**Published:** 2020-03-02

**Authors:** Rodney P Joseph, Colleen Keller, Sonia Vega-López, Marc A Adams, Rebekah English, Kevin Hollingshead, Steven P Hooker, Michael Todd, Glenn A Gaesser, Barbara E Ainsworth

**Affiliations:** 1 Center for Health Promotion and Disease Prevention Edson College of Nursing and Health Innovation Arizona State University Phoenix, AZ United States; 2 College of Health Solutions Arizona State University Phoenix, AZ United States; 3 Southwest Interdisciplinary Research Center Arizona State University Phoenix, AZ United States; 4 College of Health and Human Services San Diego State University San Diego, CA United States; 5 Edson College of Nursing and Health Innovation Arizona State University Phoenix, AZ United States; 6 Shanghai University of Sport Yangpu Qu, Shanghai Shi China

**Keywords:** eHealth, mHealth, exercise, minority health, primary prevention, heart diseases, African-American

## Abstract

**Background:**

*Smart Walk* is a culturally relevant, social cognitive theory–based, smartphone-delivered intervention designed to increase physical activity (PA) and reduce cardiometabolic disease risk among African American (AA) women.

**Objective:**

This study aimed to describe the development and initial usability testing results of *Smart Walk*.

**Methods:**

*Smart Walk* was developed in 5 phases. Phases 1 to 3 focused on initial intervention development, phase 4 involved usability testing, and phase 5 included intervention refinement based on usability testing results. In phase 1, a series of 9 focus groups with 25 AA women (mean age 38.5 years, SD 7.8; mean BMI 39.4 kg/m2, SD 7.3) was used to identify cultural factors associated with PA and ascertain how constructs of social cognitive theory can be leveraged in the design of a PA intervention. Phase 2 included the analysis of phase 1 qualitative data and development of the structured PA intervention. Phase 3 focused on the technical development of the smartphone app used to deliver the intervention. Phase 4 consisted of a 1-month usability trial of *Smart Walk* (n=12 women; mean age 35.0 years, SD 8.5; mean BMI 40 kg/m2, SD 5.0). Phase 5 included refinement of the intervention based on the usability trial results.

**Results:**

The 5-phase process resulted in the development of the *Smart Walk* smartphone-delivered PA intervention. This PA intervention was designed to target social cognitive theory constructs of behavioral capability, outcome expectations, social support, self-efficacy, and self-regulation and address deep structure sociocultural characteristics of collectivism, racial pride, and body appearance preferences of AA women. Key features of the smartphone app included (1) personal profile pages, (2) multimedia PA promotion modules (ie, electronic text and videos), (3) discussion boards, and (4) a PA self-monitoring tool. Participants also received 3 PA promotion text messages each week.

**Conclusions:**

The development process of *Smart Walk* was designed to maximize the usability, cultural relevance, and impact of the smartphone-delivered PA intervention.

## Introduction

### Background

Regular physical activity (PA) is an independent risk factor for the prevention and control of cardiometabolic diseases, including obesity, cardiovascular disease, and type 2 diabetes [1-3]. Despite the health benefits of PA, many African American (AA) women are insufficiently active (ie, do not achieve the US aerobic PA guidelines of 150 min per week of moderate-intensity PA, 75 min of vigorous PA, or an equivalent combination of durations and intensities [4]). Recent estimates from 3 national surveys measuring population-based PA indicate only 27% to 40% of AA women achieve the goals stated in national aerobic PA guidelines [5]. These low PA levels likely contribute to a high prevalence of obesity (56%) [6], cardiovascular disease (48%) [7], and type 2 diabetes (13%) [8] in AA women [9].

Emerging evidence from the proliferation of electronic health (eHealth) and mobile health (mHealth) PA interventions largely supports the preliminary efficacy of such interventions to promote PA [10-14] and improve various cardiometabolic disease risk factors [14]. A 2017 meta-analysis [15] highlighted the effectiveness of eHealth and mHealth PA interventions when compared with non–technology-delivered PA interventions. The authors examined randomized trials testing technology-delivered PA interventions (ie, interventions delivered using mobile phones, websites, social media, and email) vs non–technology-delivered PA interventions. The results indicated that technology-delivered PA interventions were 12% more effective for increasing PA than non–technology-delivered PA intervention arms. A more recent 2019 meta-analysis [16] of smartphone-delivered PA interventions further supported the use of innovative technologies to deliver PA interventions, with results showing smartphone-delivered interventions were efficient in increasing both minutes (ie, 10.5 min) and steps per day (ie, 735 steps) of PA.

eHealth and mHealth PA interventions also provide several advantages when compared with traditional face-to-face methods of intervention delivery. From a participant’s perspective, participants have the ability to access PA intervention materials virtually anywhere and at times convenient with their daily schedules. These features can help overcome barriers associated with in-person intervention delivery, including transportation issues and balancing work and family life schedules to attend intervention sessions [17-19]. From a research standpoint, technology-delivered interventions provide the opportunity for researchers to optimize theoretical fidelity (ie, defined by Rovniak et al [20] as the precision in replicating theory-based recommendations) of the behavior change principles underpinning their interventions. For example, PA interventions often use self-monitoring and feedback as methods to increase self-efficacy for PA (ie, targeting self-efficacy sources of mastery experiences and verbal persuasion). Before the development of eHealth and mHealth technologies, many researchers relied on participants to wear pedometers and manually record their PA levels in diaries. These diaries were then provided to the research team at a given time interval for review (ie, once a week or once a month) and to formulate feedback on participant progress. Current technology allows this process to occur in real time with less participant burden through the use of commercially available activity monitors, which can be viewed as a closer match to theoretical ideals. Similarly, social support for PA can be facilitated through asynchronous Web-based discussion boards, text messages, and video chats, as opposed to the traditional in-person social support sessions held at specified locations at predetermined dates and times.

Despite the favorable PA outcomes and advantages of using eHealth and mHealth approaches to deliver PA interventions, limited research has explored the efficacy of these types of interventions among AA women. This observation is surprising, given AA women engage in significantly lower PA levels than men and women of other race/ethnicities [21,22] and use electronic and mobile communication technologies (social media, internet, and mobile/smartphones) at equal or greater levels [23,24]. A recent review of eHealth and mHealth PA interventions [25] identified only 6 studies focused on AA women. Among these studies, all were preliminary in nature, and none included a smartphone-based app to promote PA (ie, 3 studies used websites, 2 used text messages, and 1 used the social media website Facebook and text messages). PA outcomes of these studies were generally favorable, with 4 (67%) reporting positive outcomes for at least one PA outcome measure. However, because of heterogeneity of study designs, multicomponent nature of interventions evaluated, and PA assessment methods, the authors were unable to determine if 1 method of intervention delivery was more effective than the another. Another main finding of this review was the lack of cultural tailoring efforts employed by researchers. Only 3 studies focusing on AA women (ie, 50%) reported some type of cultural tailoring—2 were tailored at the surface level and 1 at the deep structure level. Surface-level cultural tailoring involves matching the packaging of a health promotion intervention to the overt social and behavioral characteristics of the intended population (ie, including images of AA women and statistics regarding the PA health disparities of AA women [26]). *Deep structure* cultural tailoring involves acknowledging a group’s sociocultural values, beliefs, and behaviors and harnessing these phenomena to promote behavior change [26]. Tailoring PA interventions at the *deep structure* level may enhance the acceptability and uptake of an intervention, which is expected to lead to greater improvements in PA when compared with a nontailored or surface-tailored PA intervention.

### Objective

To address low PA levels, high cardiometabolic disease prevalence, and limitations of previous research using eHealth and mHealth technologies to promote PA among AA women, we developed *Smart Walk*, an 8-month, deep-structure culturally tailored PA intervention for AA women aged 24 to 49 years (identifier: NCT02823379 [27]). Women in this age range were included in the intervention because it allowed us to tailor the PA intervention to the sociocultural norms and lifestyle factors of adult AA women of childbearing age and coincides with the age range of adults with the highest prevalence of smartphone use [28]. The purpose of this study was to address the relative lack of published reports documenting the development process of behavioral mHealth PA interventions by describing the development and initial usability testing results of *Smart Walk.*

## Methods

### Description of the Smart Walk App

The *Smart Walk* intervention is primarily delivered through the *Smart Walk* smartphone app. This app, developed specifically for the study and available for iOS and Android operating systems, includes 4 key features: (1) personal profile pages for participants to share personal information (ie, name, picture/image, age, city/neighborhood of residence, and brief biography), (2) theory-based multimedia PA promotion modules delivered weekly through brief videos and electronic text with images, (3) asynchronous discussion boards for participants to discuss each weekly PA module and give/receive social support, and (4) a PA self-monitoring tool that integrates with Fitbit Alta HR activity monitors for participants to monitor their daily and weekly PA levels. In addition to the information delivered through the smartphone app, participants received brief PA promotion text messages delivered 3 times per week. An overview of the key features of the *Smart Walk* intervention is provided in Table 1. Screenshots of the *Smart Walk* app feature are illustrated in Multimedia Appendices 1 and 2 and in a recently published paper [29] describing the rationale and design of the *Smart Walk* intervention.

**Table 1:** Overview of *Smart Walk* intervention components.

Intervention component		Description
**Smartphone app features**		
	Personal profile pages	Comparable with personal profile pages on commercially available social media websites (ie, Twitter and Facebook) Allows participants to share information with other women in the study: name, personal picture/image, age, neighborhood/area of residence, and brief biographical narrative. Aimed at creating a sense of community among study participants.
	Weekly multimedia text and video modules	Primary delivery channel for the educational and behavioral components of the intervention. Modules consist of text- and image-based PA^a^ promotion materials and brief 3- to 7-min videos describing the PA promotion topic of the week.
	Discussion board forum with weekly discussion prompts	Companion to weekly PA promotion modules Provide a venue for participants to reflect on information presented, share their personal experiences about PA, and give/receive social support for PA. Also includes a general Community Board forum, where participants share information and/or discuss topics that may not align with the weekly module topics. Primary mechanism to foster social support for PA through dialog among participants
	PA tracker	Self-monitoring feature for tracking daily and weekly PA through interactive graphing functions Integrates with the Fitbit Alta HR activity monitor
Text messages delivered 3 times per week		Provide PA promotion reminders, tips, and encouragement

**^a^**PA: physical activity.

## Results

### Overview of the Smart Walk Development Process

**Figure 1:**
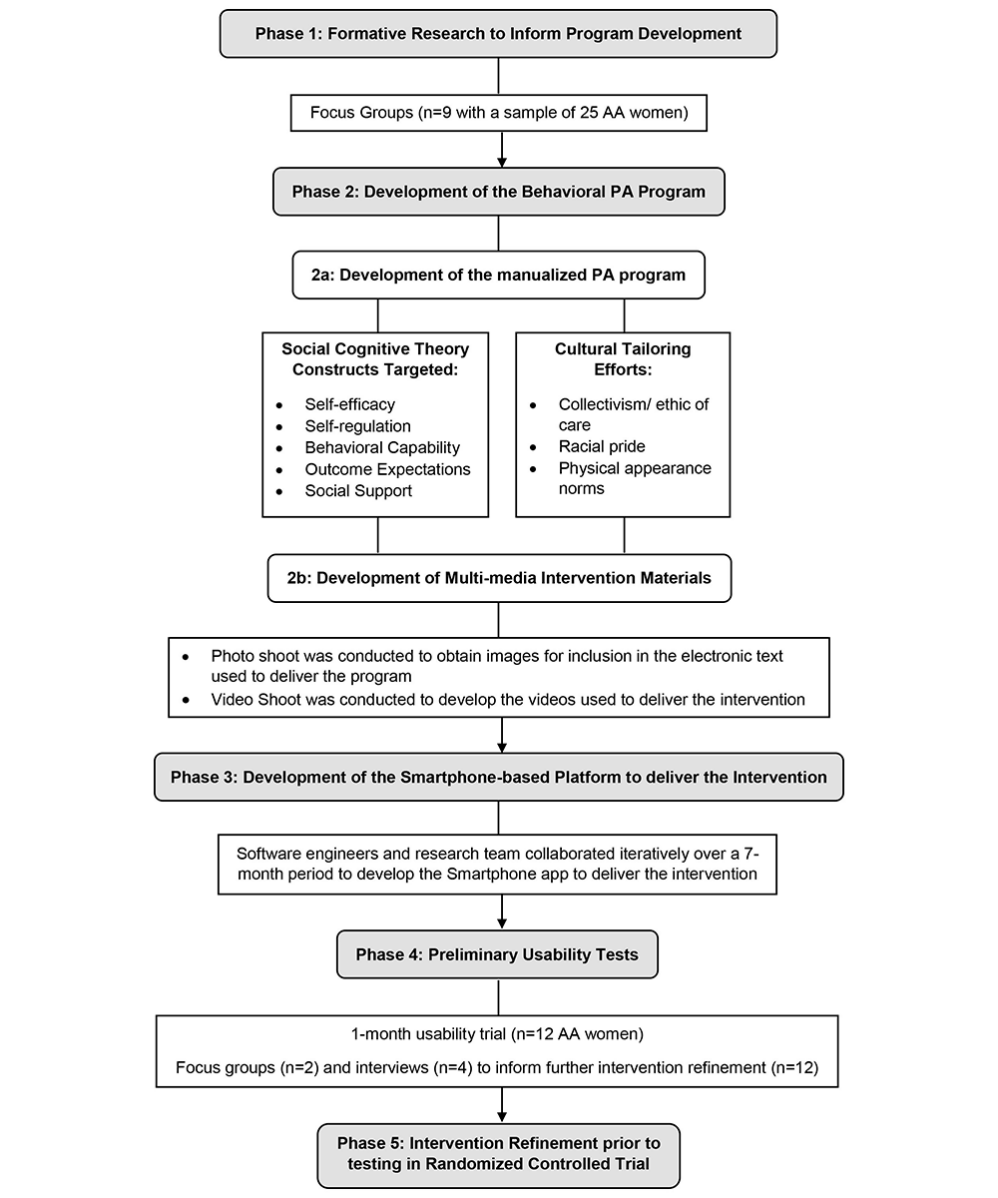
Overview of the Smart Walk development process.

The development of *Smart Walk* followed a 5-phase process. Phases 1 to 3 focused on intervention development, phase 4 involved usability testing, and phase 5 focused on intervention refinement based on usability testing results. Figure 1 provides an overview of these phases. Detailed descriptions of methods for each phase are presented below.

### Phase 1: Formative Research to Inform Theoretical and Cultural Relevance of the Smart Walk Intervention

In phase 1 of the intervention development, 25 AA women with obesity (mean age 38.5 years, SD 7.8; mean BMI 39.4 kg/m^2^, SD 7.3) were recruited to participate in a series of 3 focus group sessions. These focus groups concentrated on collecting information on AA women’s perceptions, experiences, and determinants related to PA to inform the cultural and theoretical relevance of the intervention. Eligibility criteria for participation included (1) self-identifying as an AA woman, (2) being between the ages of 24 and 49 years, (3) having a BMI greater than or equal to 30 kg/m^2^, and (4) performing less than or equal to 60 min per week of moderate-to-vigorous intensity PA (according to the 2-item Exercise Vital Sign questionnaire [30]). These inclusion criteria represented the desired target population and end users of the *Smart Walk* intervention.

The study protocol, reported elsewhere [31], engaged each participant in a series of 3 focus group sessions. Each focus group session explored specific PA-related topics to inform development of the *Smart Walk* intervention. This cohort approach allowed us to collect in-depth information from participants while also being considerate of participant burden. The results of these focus group sessions described how 5 social cognitive theory constructs (ie, self-efficacy, self-regulation, behavioral capability, outcome expectations, and social support) could be integrated in the design of a culturally relevant PA intervention for AA women. They also served to identify how cultural norms and preferences of AA women could be incorporated into the intervention design and delivery. Previously published studies [31-33] provide an in-depth description of the methodology and results of these focus group sessions.

### Phase 2: Development of the Behavioral Physical Activity Intervention and Wellness Contact Control Condition

Phase 2 of the intervention development was divided into 2 distinct subphases. The first subphase (phase 2a) focused on the development of the structured behavioral PA intervention as well as the attention-matched wellness control condition (named *Smart Health)* that would serve as a control group in the *Smart Walk* pilot trial. The second subphase (phase 2b) involved creation of the multimedia videos and print-based PA promotion materials used to deliver the PA intervention.

#### Phase 2a: Development of the Structured Physical Activity Promotion Program

In this phase, the research team used the qualitative data collected in phase 1 to refine and enhance a previously established PA promotion intervention for AA. This previously established program [34] was originally developed and pilot-tested in an 8-week randomized controlled trial using Facebook and text messages as the delivery method. Modifications to the PA program focused on (1) enhancing theoretical fidelity [20] of social cognitive theory constructs targeted by the intervention, (2) refining the cultural relevance, and (3) extending the length of the active intervention from 8 weeks to 4 months. The research team also identified and developed content for the wellness control during this phase. Health topics for the wellness control were selected based on their relevance to overall health and wellness but not inclusive of cardiometabolic disease risk (ie, breast and cervical cancer screenings, stress management, and preventing the cold and flu), including specific health topics relevant to AA women (ie, hair care and sickle cell disease). Content for the wellness contact control condition was developed through reviews of the scientific literature, rather than formative work with the study population.

Intervention content was developed collaboratively among members of the research team. These efforts resulted in the creation of 14 separate print-based PA promotion modules and 14 wellness modules to be delivered in the attention-matched control condition. Specific PA and health topics covered in these modules are presented in Table 2.

**Table 2:** Module topics by study group.

Module number	*Smart Walk* module topics	*Smart Health* module topics
1	Introduction to the national PA^a^ guidelines and the health benefits of PA	Sunscreen and skin care
2	Overview of PA-related health disparities among African American women and the importance of being a PA role model	Hydration and water consumption
3	Time management and strategies for incorporating 30 min of PA into the day	Heat illness: causes, symptoms, and prevention
4	PA goal setting	Discussing your health with your doctor
5	Overcoming general barriers to PA	Breast cancer prevention and screening
6	Tips for increasing daily PA	Cervical cancer prevention and screening
7	Overcoming hair care barriers to PA	Hair care
8	Creating a social support network for PA	Oral health
9	Trying new types of activities	Eye health
10	Reducing sedentary time	Sickle cell disease
11	Dietary behaviors to complement PA	Stress management
12	Muscle strengthening and stretching activities to complement aerobic PA	Preventing the cold/flu
13	Dealing with setbacks	Staying healthy when traveling
14	Review of previous modules and maintenance of PA after the active intervention phase	Review of previous modules

^a^PA: physical activity.

After initial drafts of the print-based PA promotion and wellness attention-matched control group materials were developed, a professional copyeditor reviewed and edited the print-based intervention materials and developed oral scripts. Edits focused on grammar, message clarity, and simplifying the text to ensure all materials were written at or below an eighth-grade reading level. Throughout the editing process, the copyeditor worked with the research team to ensure that the edits did not change the original meaning or cultural relevance of the intervention materials. Oral scripts included the same content as the print-based materials but were designed to be read out aloud by the study spokesperson during the development of the module videos used for the PA program and the wellness control condition.

Once finalized, the intervention was designed to target social cognitive theory constructs of behavioral capability, outcome expectations, social support, self-efficacy, and self-regulation and to address deep structure sociocultural characteristics of collectivism, racial pride/role modeling, and physical appearance norms of AA women (ie, hairstyle and body shape preferences of AA women). The strategies used to address these theoretical constructs and cultural characteristics are briefly described in Tables 3 and 4. Readers are also referred to a recent publication by our research team for a more in-depth description of these design characteristics [29].

#### Phase 2b: Development of the Multimedia Physical Activity and Health Promotion Modules

After the development of the structured PA promotion and attention-matched wellness control materials, the research team collaborated with a local photography/videography company to (1) create images to be included in the electronic print-based PA promotion materials and (2) develop the video vignettes used to deliver the intervention. To create the images for inclusion of the intervention materials, local AA women of diverse ages and body types were recruited to participate in a photo shoot. This photo shoot was designed to capture local AA women engaging in various types of PA. The decision to use local women in these intervention videos and images, as opposed to professional models or stock photos, was purposeful, as research [35,36] suggests that social modeling from real-life members of the community who possess characteristics of the study participants (eg, having a history of struggling with being physically active and trying to find a work and family life balance to allow being physically active) enhances the credibility and relatability of the information portrayed in a PA promotion program.

To create the 3- to 7-min intervention videos, 2 separate 1-day video shoots were conducted. At the first video shoot, initial versions of all module videos were filmed. After all videos were filmed, the videographer created rough cuts of the module videos for the research team to review. After an initial review of the videos, the research team determined several of the videos needed to be refilmed to enhance intervention delivery. After this reshoot, the videographer performed postproduction edits to all module videos and added background music, interactive graphics, and on-screen text. These postproduction edits served 2 functions: (1) to reinforce and emphasize key content discussed in the intervention and (2) to make the videos more interactive and engaging to watch. The research team worked iteratively with the videographer during this postproduction process until both parties were satisfied with final versions of all videos.

**Table 3:** Theoretical constructs targeted by the intervention.

Social cognitive theory construct	Brief description	How the construct is addressed in the intervention
Behavioral capability	Knowledge and skill to perform a PA^a^	Intervention materials provide information on the health benefits of regular PA, the national PA guidelines, how to gauge the intensity of PA performed, and strategies to achieve the national PA guidelines.
Outcome expectations	Anticipated outcomes of engaging in PA	Intervention materials highlight the health (ie, reduced cardiometabolic disease risk) and social benefits (ie, being a role model) of being physically active.
Social support	Extent to which significant referents approve, encourage, and/or influence performance of PA	Text messages provide messages of support and encouragement for PA. Discussion board forums prompt discussion focused on increasing social support for PA.
Self-regulation	Ability to manage social, cognitive, and motivational processes to achieve a desired PA goal	Participants are provided a static intervention goal of achieving a 150 min per week of PA and provided an activity monitor to self-monitor their PA to achieve this goal.
Self-efficacy	Confidence in oneself to take action and overcome barriers	Intervention messages provide encouragement for PA engagement (ie, verbal persuasion), intervention content includes images of AA^b^ women engaging in various types of PA and PA testimonials (ie, social modeling), and participants are encouraged to track their PA as they implement behavior change strategies targeted by the intervention (ie, mastery experiences).

^a^PA: physical activity.

^b^AA: African American.

**Table 4:** Cultural characteristics targeted by the intervention.

Cultural characteristics	Brief description	How the cultural characteristic is addressed in the intervention
Collectivism	Prioritization of the needs of others (ie, family/close friends) before the needs of their own, which can result in AA^a^ women reporting lack of time, energy, or resources for PA^b^ engagement. Although this phenomenon is reported among women of other races/ethnicities, previous research, including our own formative work, has suggested this concept may be more accentuated in the AA community.	Intervention materials place emphasis on the following: Importance of caretaking in the value system of AA women. Regular PA engagement is an investment in the health and well-being of AA women, not taking *time away* from their family/friends or other responsibilities. Regular PA will help AA women to perform their caretaking and other responsibilities with more energy and for a longer duration throughout the lifespan.
Racial pride/role modeling	Many AA women are aware of, and interested in, how their behaviors can contribute to the collective health and well-being of the AA community.	PA promotion materials emphasize that physically active AA women are positive role models for other members of the AA community, which can encourage others in their community (ie, family and friends) to become active and adopt healthy lifestyle behaviors.
Physical appearance preferences	Some AA women are hesitant to engage in PA because of the following reasons: Perspiration can have a negative effect on their hairstyle. They have the perception that PA will alter their desired body shape.	Intervention materials: Include hairstyling strategies to reduce the negative effects of perspiration while performing PA (ie, use hair wraps and certain hair care products that negate the effects of sweating) and encourage women to adapt hairstyles that are less affected by perspiration (ie, braids and natural hairstyles). Inform participants that engaging in PA at the levels recommended by the study (ie, 150 min per week) will not substantially change their body shape unless they also change their dietary habits. Emphasize the health benefits of PA independent of weight loss (ie, reduced cardiometabolic disease risk, weight maintenance, and increased energy).

^a^AA: African American.

^b^PA: physical activity.

### Phase 3: Development of the Smartphone-Based Platform to Deliver the Physical Activity Program

Phase 3 of program development focused on technical development of the smartphone app platform used to deliver the interventions. This task was accomplished through a collaborative process involving the study’s principal investigator (PI) and a software engineer serving on the project team.

During initial meetings with the software development team, the PI provided a general overview of the operating system requirements (ie, available for iOS and Android devices), desired features, and preliminary thoughts on the visual layout of the study apps. Drawing on these initial discussions, the software engineer worked in 1- to 2-week iterative *sprints* over the course of a 7-month period to build the platform used to deliver both the PA and wellness attention-matched control interventions. During each sprint, the software engineer worked on a defined task until a prototype was ready for preliminary review and usability testing by the research team. Following review and testing by the research team, either further modifications were requested or the prototype was approved and filed as *complete*. This process was repeated over the course of 7 months until an initial version of both smartphone apps was developed.

The initial prototype of the *Smart Walk* intervention included the following 3 features: (1) multimedia PA promotion modules consisting of brief videos and electronic text with images, (2) discussion boards for participants to discuss the weekly PA modules and give/receive social support, and (3) a PA self-monitoring/tracking tool that integrated with the Fitbit Alta HR activity monitor. The *Smart Health* app included all the same features, with the exception of the PA self-monitoring/tracking feature. Screenshots of these initial prototypes are available upon request from the first author of the paper.

### Phase 4: Preliminary Usability Test Trial

After the initial development of the study smartphone apps, a 1-month usability trial was conducted. The methods used in this trial allowed the research team to obtain participant feedback on (1) specific issues associated with the usability and functionality of the study apps and (2) their overall thoughts regarding health promotion programs when implemented in the real world. This trial also provided the opportunity for the research team to test the computer-based algorithms used to deliver the interventions (ie, uploading of new modules and discussion board topics and delivery of text messages) before large-scale testing.

#### Usability Trial Methods

A total of 12 insufficiently active (ie, ≤60 min per week of at least moderate PA according to the Exercise Vital Sign questionnaire [30]) AA women with obesity (ie, BMI≥30 kg/m^2^) who were between the ages of 24 and 49 years participated in this trial. These sample characteristics reflect the inclusion criteria for women subsequently recruited for a randomized pilot trial of the intervention. Participants were randomly assigned using stratified randomization based on participants’ smartphone operating system (ie, iOS or Android) to receive either the culturally relevant PA program (ie, *Smart Walk)* or the wellness attention-matched control (*Smart Health*). This randomization method ensured balanced feedback from users of both operating systems on both study apps. After randomization, participants received all intervention materials originally designed to be delivered over a 4-month active intervention period in an abbreviated 1-month trial period.

During the trial, study staff communicated with participants (ie, via telephone, text message, or email based on participants’ preferences) to inquire about any functionality or usability issues with the apps during weeks 1 and 3. Participants also occasionally emailed study staff and/or posted comments on the app discussion boards when they experienced a usability or functionality issue. After completion of the 1-month trial, participants were invited to participate in a focus group session to provide feedback on the smartphone-delivered program they received. Women not available to participate in the focus group session were provided the option to participate in a one-on-one in-person interview. Participants were provided US $30 for participating in the 1-month demonstration trial and an additional US $20 for participating in a focus group or interview session. No other strategies for recruitment or retention were employed.

Focus groups and interviews were led by an AA facilitator who was trained in qualitative data collection methods by the study PI. Guides used to facilitate focus group and interview sessions are presented in Textbox 1. All data collection sessions were audio-recorded, transcribed verbatim, and imported into NVivo12 (QSR International) for analysis. Content analysis [37] was used to analyze the participant narratives. In total, 2 coders reviewed study data independently and then met to discuss themes until consensus was reached. Final themes used in the analysis were based on major topics explored by the focus group/interview guide and repetitive themes that emerged during data collection assessments.

**Postusability trial focus group/interview guide questions. Similar guides were used in both study arms. The only differences between guides were (1) the reference to either *Smart Walk* or *Smart Health*, and (2) question 6 was only asked for participants assigned to the *Smart Walk* study group.**

1. What are your overall thoughts about the [Smart Walk or Smart Health] app?
2. Please share your thoughts about the overall usability and functionality of the [Smart Walk or Smart Health] app.
3. Thinking about the multimedia video and text modules on the [Smart Walk or Smart Health] app, what are your thoughts on them?
4. What are your thoughts about the text messages you received?
5. Please describe your thoughts about the [Smart Walk or Smart Health] Discussion Boards?
6. Let’s talk about the activity tracking feature. What are your thoughts on using it?
7. Overall, what are your opinions on the [Smart Walk or Smart Health] program as whole?
8. Other than a smartphone application, how would you like to receive a health promotion program?
9. Is there anything else you would like to tell us about the Smart Walk [or Smart Health] application or physical activity program that we have not already discussed?

#### Usability Trial Results

The 12 participants had a mean age of 35.0 years (SD 8.5) and a mean BMI of 40 kg/m^2^ (SD 5.0). Of these 12 participants, 7 accessed the study smartphone apps with an Android device (n=3 in PA arm and n=4 in wellness arm), and 5 accessed the study smartphone apps with an iOS device (n=3 in PA arm and n=2 in wellness arm). Feedback regarding the usability and functionality of the *Smart Walk* app was obtained through 2 focus group sessions (group sizes were n=4 and n=2). Feedback on the *Smart Health* app was collected through 1 focus group (n=2) and 4 one-on-one interviews. The qualitative assessments lasted between 33 and 56 min for the focus group sessions and between 18 and 22 min for the one-on-one interviews. Participant narratives were classified into 3 overarching themes: usability/functionality issues, desire for enhanced personalization, and overall impressions of the intervention.

#### Usability/Functionality Issues

Participants identified several usability and functionality issues for the *Smart Walk* and *Smart Health* study apps. These concerns were classified into subthemes according to specific app feature. Participant quotes illustrating usability/functionality issues discussed below are presented in Multimedia Appendix 3, as are the refinements made to the intervention based on participant feedback.

#### Multimedia Modules

Overall, participants in both study groups reported encouraging sentiments regarding the information delivered through the multimedia modules. However, several usability and functionality issues associated with module delivery emerged. These included (1) excessive video buffering when participants had limited cellular service and/or intermittent wireless internet network connection; (2) audio portion of videos was not playing on select Android devices (this was because of an error in video filter settings); and (3) video display on iOS devices defaulting to full screen, which did not allow participants to simultaneously listen to the video and read the text.

#### Discussion Board Forums

Participants identified 2 key usability/functionality issues associated with the app’s discussion board feature. The first was a platform-specific issue for some, but not all, Android users that resulted in a deletion of unsaved text if participants rotated their phone while typing a discussion board post. The second issue was related to participant desire for enhanced interactivity. Participants indicated that they would like to receive real-time notifications when other study participants posted on the discussion boards, as opposed to having to periodically check the study app to see if anyone posted and/or replied to the boards. When discussing strategies to provide these notifications, text messages and push notifications were mentioned by participants. As the conversations progressed, it became clear that most participants preferred the use of push notifications over text messages.

#### Activity Tracker

Participant feedback on the activity tracker feature revealed 2 primary concerns. The first issue was related to the wrist-worn Fitbit activity monitor not accurately recording all moderate-to-vigorous activities. Several participants noted engaging in physical activities with restricted arm movement (ie, stationary cycling) that were not accurately being recorded by the wrist-worn device. Similarly, a few participants perceived this to be an issue, when in actuality, they were engaging in activities not considered moderate-to-vigorous intensity aerobic activities (ie, yoga and walking at a cadence that did not meet the criterion for moderate-intensity activity). The second issue was that, for some participants, the commercial Fitbit software was not automatically communicating with the *Smart Walk* app. This technological issue resulted in the *Smart Walk* app not always updating participants’ minutes per day of moderate-to-vigorous PA unless they first opened and refreshed the commercial Fitbit app.

#### Desire for Enhanced Personalization and Individual-Level Tailoring

Throughout the focus groups and individual interviews, participants expressed the desire for both study smartphone apps to include additional features to promote an enhanced sense of personalization and individual-level tailoring. Participants described that although they viewed the intervention content as favorable, additional steps could be taken to facilitate a sense of community among study participants and to individually tailor the program to each participant. Discussions on this topic resulted in participants suggesting 3 key modifications to the study smartphone apps: (1) addition of personal profile pages, (2) personalization of the discussion boards, and (3) individual-level tailoring of text messages. Participant quotes highlighting these suggested revisions to the study app are presented in Mutlimedia Appendix 3.

#### Addition of Personal Profile Pages

One strategy that seemed to enhance personalization of the apps was the addition of a personal profile feature. This feature was initially brought up in the first *Smart Walk* focus group session. Subsequent conversations regarding the creation of personal profile pages revealed that participants envisioned this feature to be similar to profile pages on social media websites (ie, Facebook and Twitter) and expressed that adding this feature would help create a stronger sense of community among users. Specific information recommended by participants to include on this feature included participant name, personal picture/image, city/neighborhood of residence, and a brief biography. However, it should be noted that not all participants were as enthusiastic about the idea of adding a personal profile page to the study apps. In the end, there was a consensus among participants that the profile page would be a good addition if it was optional for participants to complete.

#### Personalized Discussion Board Forums

Another topic that emerged was participants’ desire for additional discussion board forums where they could create and/or drive the conversation narrative, rather than relying on the weekly topic-specific discussion board prompts designed to facilitate discussion. Discussion board forums during the usability trial were tied directly to the weekly module topics and included prompts to facilitate topic-specific discussion. Some participants alluded to the notion that this felt restrictive and did not provide an opportunity to engage in organic dialog on topics not directly related to the weekly module topics. Participants indicated that they would like the opportunity to create their own discussion threads and discuss topics not specifically related to the weekly modules.

#### Individual-Level Tailoring of Text Messages

Discussion on the study text messages revealed 2 minor modifications that could be implemented to help achieve a sense of individual-level tailoring: (1) address participants by name in the text messages and (2) allow participants to specify the time of day they would like to receive text messages. Several participants noted that they perceived the text messages as impersonal or generic. These women emphasized that including their name in the text messages would make them feel *special* and provide a sense of individual-level tailoring. With reference to the time of day the text messages were delivered, the study protocol during the demonstration trial had all participants receive a text message at 8:30 am. Several participants stated that this time was not ideal, and they would prefer to select the time of day to receive the text messages.

#### Overall Impressions of the Intervention

Participants reported favorable overall impressions of the smartphone-delivered interventions. Quotes highlighting this sentiment for participants assigned to the PA intervention (ie, *Smart Walk*) included, “The app itself, I thought it was really informative, and it was fun to watch the videos, and participate in the discussion boards,” “I learned new stuff,” and “I thought that it was cool... And it was clear. So I enjoyed it.” Similarly, participants assigned to the overall health and wellness group (ie, *Smart Health*) enjoyed the app and felt it was useful for others. Despite favorable overall impressions of the intervention, several participants noted the abbreviated 1-month duration of the trial resulted in too much information being delivered over a short period (ie, 1-2 text messages each day and new module and discussion board prompt every 2-3 days). Quotes highlighting this included “I think the text messages were a bit excessive.” and “Since it was condensed, we go text more often than we normally would...I had to remember that. I’m like, this is a little, I can’t keep up.” As conversations on this topic continued and the facilitator described the delivery schedule for forthcoming randomized controlled trial (ie, 1 new module topic/discussion board prompt per week and 3 text messages each week during the active 4-month intervention phase), participants expressed that the intervention dose for which materials were originally designed to be delivered seemed more appropriate.

### Phase 5: Intervention Refinement

Phase 5 focused on refining the intervention according to participant feedback from phase 4. To accomplish this, the research team systematically evaluated participant narratives and recommendations for intervention improvement. On the basis of this analysis, numerous refinements and modifications were made to the study apps, which are illustrated in Multimedia Appendix 3. The screenshots of the final versions of both the *Smart Walk* and *Smart Health* apps are presented in Multimedia Appendices 1 and 2.

## Discussion

### Principal Findings

The development of the *Smart Walk* interventions occurred over a 3-year period. It was made possible through a National Institutes of Health (NIH) career development award (ie, K99HL129012 and R00HL129012) and involved expertise from a diverse set of researchers, software engineers, industry professionals (ie, videographer/photographer and copyeditor), and intended users of the intervention. The methods used emphasize the importance of creating a transdisciplinary research team when developing innovative approaches to address major public health issues and the significance of conducting extensive formative research with end users of an intervention.

A novel aspect of the information reported in this paper is the methods and results of the 1-month usability trial (ie, phase 4 of intervention development). The methods employed in this phase allowed the research team to obtain feedback on the usability and functionality of the study apps as well as participants’ overall impressions of the interventions when implemented in a real-world setting. We selected this method of participant usability testing, as opposed to more preliminary laboratory-based methods (ie, user-centered design or *think aloud* techniques), because the research team conducted these types of laboratory tests internally during phase 3 of intervention development. Given the research team included commercial mHealth app users who also have experience developing research-based eHealth and mHealth PA interventions [34,38,39], we relied on our previous experience and expertise in these areas to conduct preliminary usability tests.

The results of the usability trial emphasize the importance of pilot-testing smartphone apps on various operating systems (ie, iOS and Android) and smartphone devices, as our results indicated app functionality varied based on these factors (ie, video sound was not audible on select Android devices and defaulted to full screen on iOS devices). Another important aspect that emerged from the usability trial was the need to enhance the individual-level tailoring and/or personalization of the interventions. Participants discussed, at length, the need for the interventions to be tailored to them at the individual level and not just to their sociocultural characteristics associated with being an AA woman. The strategies proposed by participants to achieve this level of personalization included incorporating their names into the app features (ie, home page and activity tracker) and text messages, allowing them to create personal profiles, and creating a community discussion board forum where they can drive the discussion narrative, rather than the research team. Participants also noted the need to enhance the interactivity of the app discussion boards by adding push notifications to notify participants when other women post on the discussion boards. Participants described that this feature is common among other apps they use (ie, Facebook) and is expected if a goal of the study was to promote communication and dialog with other users.

The results of the usability trial also highlight the challenges researchers face when developing mHealth interventions. As evidenced by our usability trial results, participants expect research-based smartphone apps to be just as interactive, engaging, and easy to use as commercially available apps. In our experience, this can be somewhat difficult to achieve because of grant funding cycles (ie, typically no more than 2-5 years to develop, implement, and evaluation outcomes), available resources (ie, money and access to software engineers), and the rapid pace at which technology changes. In addition, there is a need for researchers to justify why creation of a new app is necessary, as there are thousands of other health promotion apps commercially available to consumers. In our case, the novelty of the intervention is that it is driven by theory and deep structure and culturally tailored to the sociocultural norms and behavior preferences of AA women, which is an important addition of the eHealth and mHealth field of study. These factors are designed to enhance acceptability and salience of the intervention, which, in turn, is expected to lead to the desired behavioral outcome of increased PA.

It is also important to discuss the nuanced relationship between the behavioral PA program and the smartphone app used to deliver it, as we view these as complementary yet separate entities. The behavioral PA program, developed in phases 1 and 2, can be adapted for delivery using other modalities (eg, in-person group-based sessions) without significantly altering cultural relevance or theoretical underpinnings of the program. Similarly, the smartphone app used to deliver the PA program can be modified to deliver other health promotion programs (ie, as was done for the *Smart Health* attention-matched wellness control condition). Given the differentiation between the behavioral PA program and the delivery channel can sometimes be a source of confusion when discussing eHealth and mHealth research with colleagues, community members, and students alike, we encourage researchers conducting future eHealth and mHealth interventions to clearly distinguish between the behavioral components of a program and the delivery channel when applicable.

### Future Directions

*Smart Walk* is currently being tested in an 8-month randomized controlled pilot trial. Cardiometabolic end points of the intervention include reductions in total cholesterol, low-density lipoprotein cholesterol, systolic and diastolic blood pressure, fasting glucose, aortic pulse wave velocity, and proinflammation cytokines (tumor necrosis factor alpha and interleukin 1 beta); an increase in high-density lipoprotein cholesterol; and improvements in cardiorespiratory fitness.

Data collected from this trial will provide information on preliminary efficacy of the intervention and highlight areas for further intervention refinement before large-scale testing.

### Conclusions

*Smart Walk* is an innovative approach to promote PA and reduce cardiometabolic disease risk among AA women. The formative work described in this paper was designed to enhance the cultural and theoretical relevance of the intervention as well as optimize user experience with using the Smart Walk app. The methods described can provide a framework for other researchers to follow when developing future eHealth and mHealth interventions.

## Appendix

Multimedia Appendix 1 Screenshots of the Smart Walk smartphone app [28].

Multimedia Appendix 2 Screenshots of the Smart Health smartphone app [28].

Multimedia Appendix 3 Modifications and refinements to the intervention based on usability trial results.

## Abbreviations

AA: African American
eHealth: electronic health
mHealth: mobile health
NIH: National Institutes of Health
PA: physical activity
PI: principal investigator
