# Supplementary material for: A Culturally Relevant Smartphone-Delivered Physical Activity Intervention for African American Women: Development and Initial Usability Tests of Smart Walk
Source: JMIR Mhealth Uhealth. 2020 Mar 2;8(3):e15346. doi: 10.2196/15346 (PMC7076402; doi:10.2196/15346)
Supplement: Multimedia Appendix 1 [file mhealth_v8i3e15346_app1.pdf]

Panel A

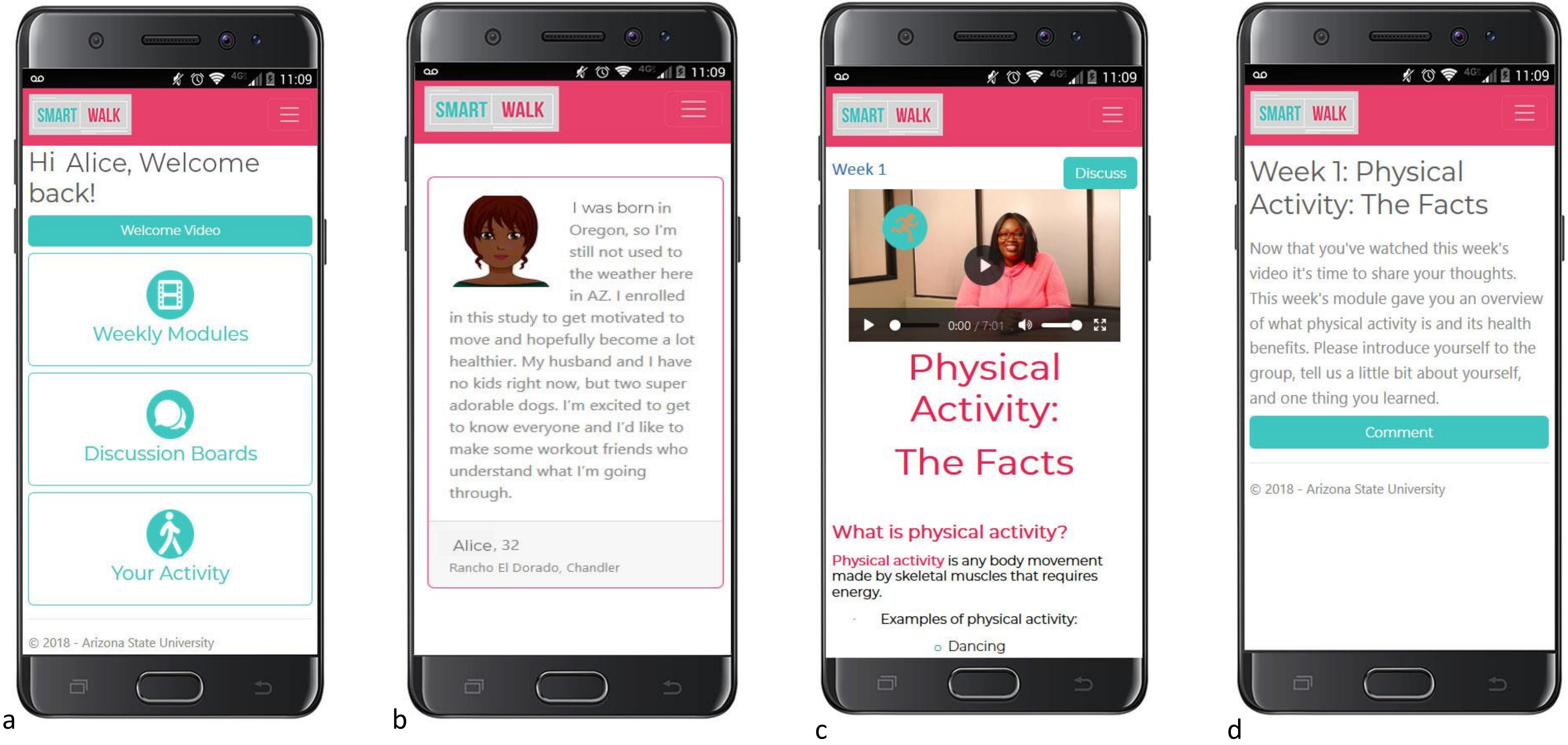

Panel A: (a) Home screen. (b) Example personal profile page, (c) Example multi-media module, (d) Example discussion board prompt.

Panel B

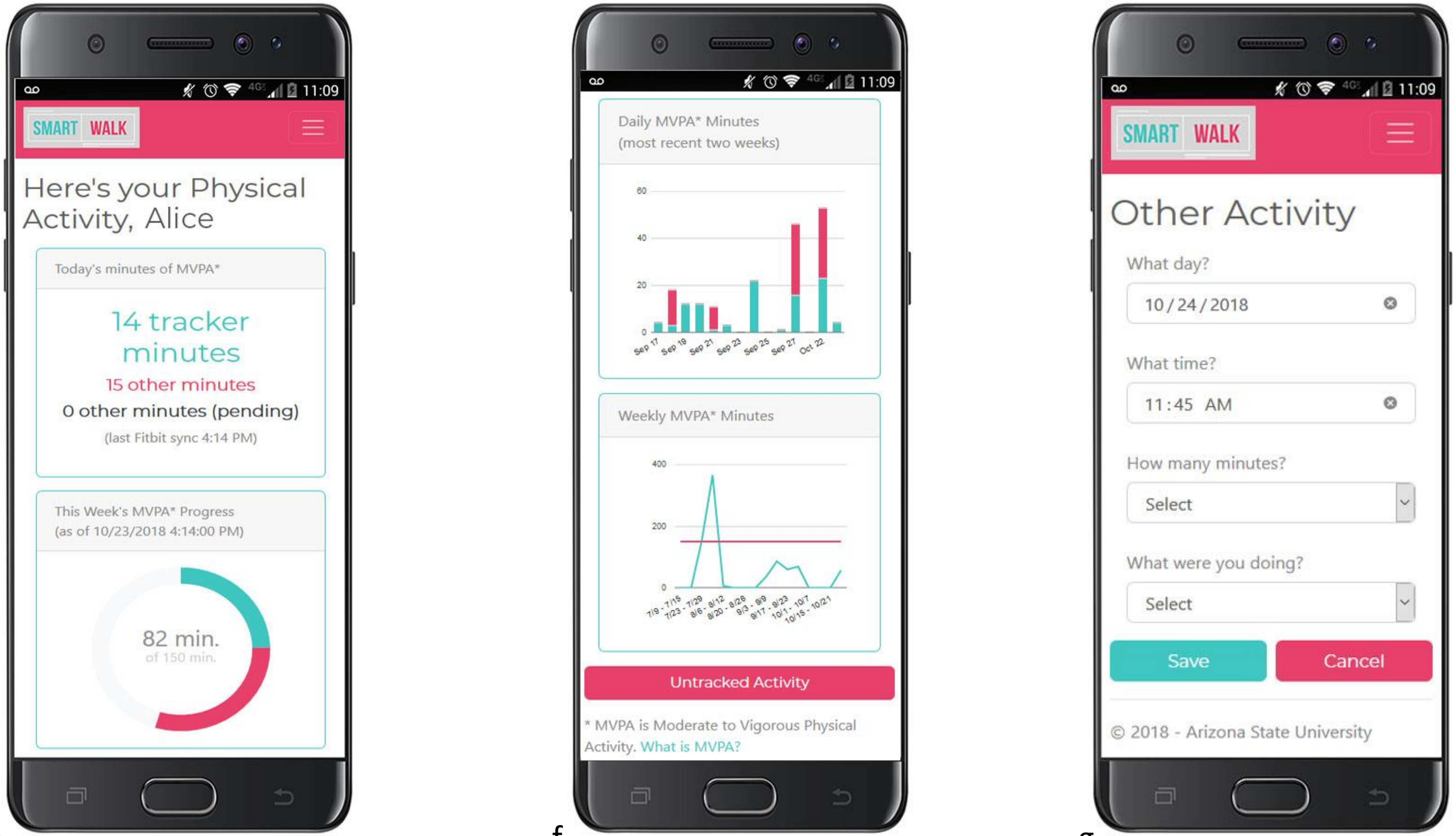

e f g

Panel B: (e) Upper box: Illustration of how daily minutes of MVPA are displayed in the activity tracker; Lower box: Illustration of donut graph used to show progress towards the weekly goal of 150 minutes of at least moderate-intensity PA. (f) Upper graph: Illustration how daily minutes of MVPA are displayed over a period of 2 weeks; Lower graph: Illustration of weekly PA levels over a 4 week period, pink line represents 150 minutes of MVPA. (g) Illustration of how non-Fitbit measured MVPA is entered into the tracking feature.
