# Supplementary material for: A Culturally Relevant Smartphone-Delivered Physical Activity Intervention for African American Women: Development and Initial Usability Tests of Smart Walk
Source: JMIR Mhealth Uhealth. 2020 Mar 2;8(3):e15346. doi: 10.2196/15346 (PMC7076402; doi:10.2196/15346)
Supplement: Multimedia Appendix 2 [file mhealth_v8i3e15346_app2.pdf]

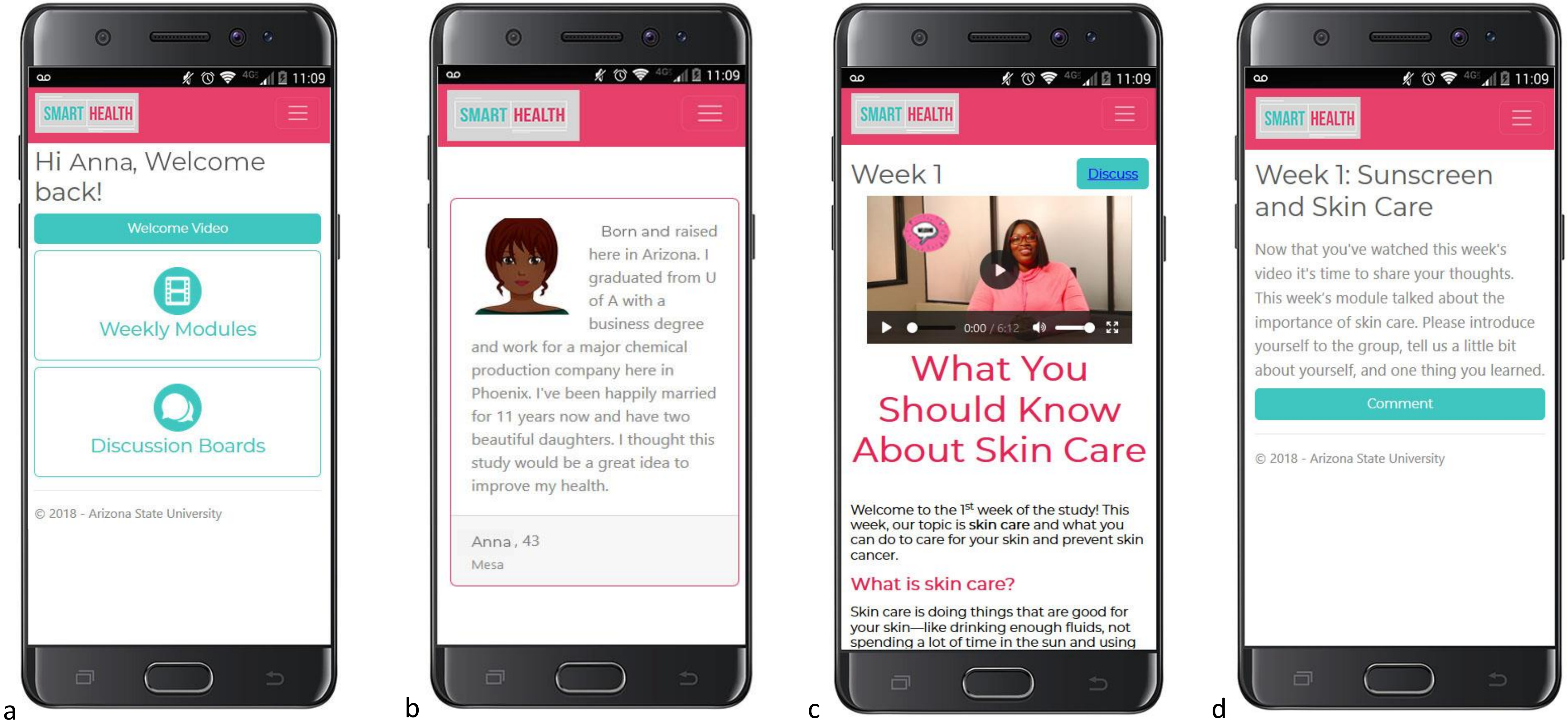

(a) Home screen. (b) Example personal profile page, (c) Example multi-media module, (d) Example discussion board prompt.
