# Supplementary material for: A Culturally Relevant Smartphone-Delivered Physical Activity Intervention for African American Women: Development and Initial Usability Tests of Smart Walk
Source: JMIR Mhealth Uhealth. 2020 Mar 2;8(3):e15346. doi: 10.2196/15346 (PMC7076402; doi:10.2196/15346)
Supplement: Multimedia Appendix 3 [file mhealth_v8i3e15346_app3.docx]

| Focus group theme | Intervention component targeted | Topic of issue/concern noted by participants | Example participant quote illustrating issue | Modification made to intervention |
| --- | --- | --- | --- | --- |
| Usability/functionality issues | Modules | Excessive video buffering | “The only issues that I have was the technological issues with the videos and there was a couple of times where it just buffered.”  “There was couple times where they [the videos] just buffer and nothing happened…” | Video hosting was moved from a university-owned server to a commercially owned server that automatically adjusts video resolution based on participant wireless connection speed |
|  | Modules | Video sound not working on select Android devices | “I could see the person speaking, but couldn’t hear anything.” | Video filter settings were reviewed for all study videos; videos with incorrect sound filter settings were corrected |
|  | Modules | Video play on iOS devices defaulted to full screen, which did not allow participants to listen to videos while also reading the module text | “I didn’t realize that pretty much everything she said in the video was in the text beneath the video, because the way it showed up on my screen, it was just a video.” | Video display settings were modified so that participants could play the videos while also reviewing module text |
|  | Discussion boards | If an Android user rotated their phone while typing a response to a discussion board post, the text field reset and deleted the text previously entered | “If I’m typing all my answers to the discussion, and then my phone turns and it’s all gone, and I have to start over.” | Programming error was resolved to alleviate this problem |
|  | Discussion boards | Lack of notification when another user commented/posted on the discussion boards | “The one thing that I would like to add is that once someone responds to a discussion board that others receive a prompt or a notification so that we know when someone else posted so we can look at it and respond to it. Because I think it would have brought more attention to make the discussion more fluid.” | Push notifications were added to the app to notify participants of when a new post was added to the discussion boards; a daily email digest was included so that a daily email notification was sent to participants illustrating unread message board threads |
|  | Activity tracker | Participants engaged in activities not accurately collected by the wrist-worn Fitbit device or engaged in activities that were not considered moderate-intensity aerobic activities | “The first week I did hot yoga and I was slowly dying, sweating, hot, heart racing and I thought, yeah, same thing, I was like, ‘Sweet! I made it’. And then I opened it and it was zero and I was like, ‘What? There’s no way’.” | An additional feature was added to the activity tracker so that participants could manually enter activities performed that are not accurately captured by the wrist-worn Fitbit device. This feature prompts researchers to either *approve* or *reject* the activity. Rejected activities are accompanied with a note describing why the activity is not considered an aerobic activity. |
|  | Activity tracker | Fitbit commercial app not communicating with *Smart Walk* app; required some participants to refresh the Fitbit before activity would display on *Smart Walk* app | “Sometimes it doesn’t update right away. So then I, from there, kept going into Fitbit to make sure I’m clicking down on that and letting it register the steps, then hoping that it’s talking to the app.” | Programming issue that resulted in this occurrence was resolved |
| Desire for enhanced personalization/individual-level tailoring | App home screen, activity tracker, text messages | App features and text messages did not include participant name | “I want you to say my name so I can feel a certain type of way about it.”  “I think it [addressing participants by name] creates a sense of intimacy, so that you feel like it’s customized towards you.” | - App home screen was updated to include the following greeting: “Welcome [insert participant name]” - Activity tracker page includes the following salutation: “Here’s Your Physical Activity [insert participant name]” - Text messages judiciously integrate participants’ names when appropriate |
|  | App home screen | Lack of personal profile feature | “Would it be a possibility for us to be able to create a profile? ... Where it’s like, “Hi, I’m [participant name redacted]. I’m 29 and this is my first time trying to figure out this journey.” I think it adds a more human aspect to it...” | Inclusion of profile page that allows participants to share the following with other study members:   - Picture - Name - Age - City/neighborhood they reside - Brief biography |
|  | Text messages | Participants wanted to select a specific time to receive study text messages | “I like the idea of the text messages. I just want to be able to say when is the best time to deliver them, when I’m actually going to look at it.” | Text message are sent at a participant-specified time collected during the baseline assessment period |
|  | Discussion board | Women desired a separate discussion board where they could drive the narrative and/or topic discussed | “I think it would be neat if in addition to the structured discussion board, there was a general discussion board...just if you wanted to talk to somebody. Just, ‘Hey, I’m struggling this week. How y’all doing?’ Something were it’s not necessarily controlled by you guys, but we control that narrative.” | A new discussion board feature was added, titled “Community Board” where participants can create threads and discussion topics not directly related to the weekly module topics. |
